# High-dose methylprednisolone mediates YAP/TAZ-TEAD in vocal fold fibroblasts with macrophages

**DOI:** 10.21203/rs.3.rs-4626638/v1

**Published:** 2024-07-19

**Authors:** Ryosuke Nakamura, Renjie Bing, Gary J. Gartling, Michael J. Garabedian, Ryan C. Branski

**Affiliations:** NYU Grossman School of Medicine; NYU Grossman School of Medicine; NYU Grossman School of Medicine; NYU Grossman School of Medicine; NYU Grossman School of Medicine

**Keywords:** voice, larynx, vocal fold, inflammation, steroids, glucocorticoids, fibroblasts, macrophages

## Abstract

The pro-fibrotic effects of glucocorticoids may lead to a suboptimal therapeutic response for vocal fold (VF) pathology. Targeting macrophage-fibroblast interactions is an interesting therapeutic strategy; macrophages alter their phenotype to mediate both inflammation and fibrosis. In the current study, we investigated concentration-dependent effects of methylprednisolone on the fibrotic response, with an emphasis on YAP/TAZ-TEAD signaling, and inflammatory gene expression in VF fibroblasts in physical contact with macrophages. We sought to provide foundational data to optimize therapeutic strategies for millions of patients with voice/laryngeal disease-related disability. Following induction of inflammatory (M(IFN/LPS)) and fibrotic (M(TGF)) phenotypes, THP-1-derived macrophages were seeded onto HVOX vocal fold fibroblasts. Cells were co-cultured +/−0.3–3000nM methylprednisolone +/− 3μM verteporfin, a YAP/TAZ inhibitor. Inflammatory (*CXCL10, TNF, PTGS2*) and fibrotic genes (*ACTA2, CCN2, COL1A1*) in fibroblasts were analyzed by real-time polymerase chain reaction after cell sorting. Ser211-phosphorylated glucocorticoid receptor (S211-pGR) was assessed by Western blotting. Nuclear localization of S211-pGR and YAP/TAZ was analyzed by immunocytochemistry. Methylprednisolone decreased *TNF* and *PTGS2* in fibroblasts co-cultured with M(IFN/LPS) macrophages and increased *ACTA2* and *CCN2* in fibroblasts co-cultured with M(IFN/LPS) and M(TGF). Lower concentrations were required to decrease *TNF* and *PTGS2* expression and to increase S211-pGR than to increase *ACTA2* and *CCN2* expression and nuclear localization of S211-pGR. Methylprednisolone also increased YAP/TAZ nuclear localization. Verteporfin attenuated upregulation of *CCN2*, but not *PTGS2* downregulation. High concentration methylprednisolone induced nuclear localization of S211-pGR and upregulated fibrotic genes mediated by YAP/TAZ activation.

## INTRODUCTION

The vocal fold (VF), an essential apparatus for phonation, vibrates hundreds of times a second.^1^ Due to its role and anatomical location, the VF is inherently exposed to mechanical and environmental stress. As many as 20 million people report voice disorders annually in the US.^2^ Inflammation is broadly associated with dysphonia and is likely etiologic for benign vocal fold lesion development. Glucocorticoids (GCs) are frequently administered to manage voice disorders because of their anti-inflammatory functions and affordability.^3–6^ However, recent reports suggest clinical outcomes of GC therapy to be variable.^7–9^ Diverse functions of GCs likely underlie the disparate outcomes.^10–12^ Of note, fibrotic effects of GC signaling have been reported in several organs and VF fibroblasts.^13–15^ Optimizing GC therapy to minimize fibrosis while limiting inflammation has the potential to benefit millions of patients.

In the inflammatory milieu, dysfunctional cooperation between tissue-resident and infiltrated hematopoietic cells can drive pathological tissue responses, such as chronic inflammation and fibrosis.^16^ Previous studies revealed the significance of intercellular communication between fibroblasts and macrophages in pathologies across multiple organs, including the VF.^17–20^ Differential macrophage phenotypes are induced via exposure to stimuli through the shift from inflammatory to fibrotic environments.^17,21,22^ Inflammatory stimuli, such as interferon-gamma (IFN- γ) and lipopolysaccharide (LPS), induce the inflammatory M1 phenotype.^17,23^ Interleukin (IL)-4, IL10, and transforming growth factor-β (TGF-β) drive the anti-inflammatory/fibrotic M2 phenotype.^23–25^ However, various subtypes beyond the dualistic classification to the M1 and M2 are induced by individual stimuli,^17^ likely related to organ-specific responses to macrophages.^26,27^ For example, in VF fibroblasts, fibrotic genes were not activated by paracrine signaling from IL4-stimulated typical M2 macrophages,^27^ which elicited a fibrotic response in non-VF fibroblasts.^28,29^ Conversely, TGF-β-stimulated macrophages induced a fibrotic response in VF fibroblasts. Independently, physical contact and paracrine signals from macrophages differentially activated VF fibroblasts.^27^ To that end, understanding interactions between VF fibroblasts and macrophages is foundational to optimally treat VF disease.

Recently, refinement of GC dose has emerged as a possible strategy to improve GC therapy.^15,30^ Our previous work with indirect co-culture models found fibrotic and inflammatory responses of VF fibroblasts triggered by macrophage-derived paracrine signals were promoted and inhibited by ‘high’ and ‘low’ concentrations of methylprednisolone, respectively.^31^ Based on this finding, we hypothesized minimizing GC concentrations to sufficiently inhibit inflammation improves efficacy of GC therapy. However, previous co-culture studies employed a cell culture insert to allow only paracrine signaling. Considering the *in vivo* environment in which macrophages directly engage fibroblasts,^32^ co-culture models with direct intercellular communication further support the translation of *in vitro* findings to support *in vivo* investigation. In addition, mechanisms underlying concentration-dependent negative and positive gene regulation remain unknown.

Based on the currently known biochemistry of GC signaling, unrelated to concentration-dependency, complex reactions of the GC receptor (GR) are thought to be a source of diverse GC functions.^10,33^ GR interacts with numerous proteins. The GC/GR complex binds and inhibits other transcription factors in the cytoplasm. Alternatively, GR translocated to the nucleus binds to both negative and positive gene regulatory elements. Various post-translational modifications (phosphorylation, acetylation, SUMOylation) are involved in GR distribution and recruitment to gene regulatory elements. Additionally, accessibility to negative and positive gene regulatory elements is putatively altered by dimerization of GR concentrated in the nucleus.^10^

Despite diversity of GR interactions with other signaling pathways,^11^ recent transcriptomic analysis on VF fibroblasts highlighted the impact of GR on the Hippos signaling pathway, which has a key role in fibrosis.^34–37^ In this pathway, Yes-associated protein (YAP) and transcriptional co-activator with PDZ-binding motif (TAZ) are the core.^38^ Activated YAP/TAZ enters the nucleus and primarily serves as co-activators of TEA domain transcription factors (TEADs) to induce TEAD-dependent transcription. *CCN2*, a fibrotic gene induced by high-concentration GCs, is a target of YAP/TAZ-TEAD signaling;^39^ this finding underlies the hypothesis that YAP/TAZ-TEAD signaling is specifically activated by high-concentration GCs.

In the current study, a direct co-culture model was employed to further confirm concentration-dependent effects of methylprednisolone to alter fibrotic and inflammatory responses of human macrophages and VF fibroblasts. We additionally explored nuclear localization of GR and YAP/TAZ in this model to interrogate mechanisms underlying concentration-dependent effects of GCs. Ultimately, we seek to refine GC therapy corroborated by mechanistic insight, to benefit millions of patients with voice-related disability.

## RESULTS

### Methylprednisolone altered inflammatory genes in direct co-culture of human VF fibroblasts and macrophages

**Figure 1:**
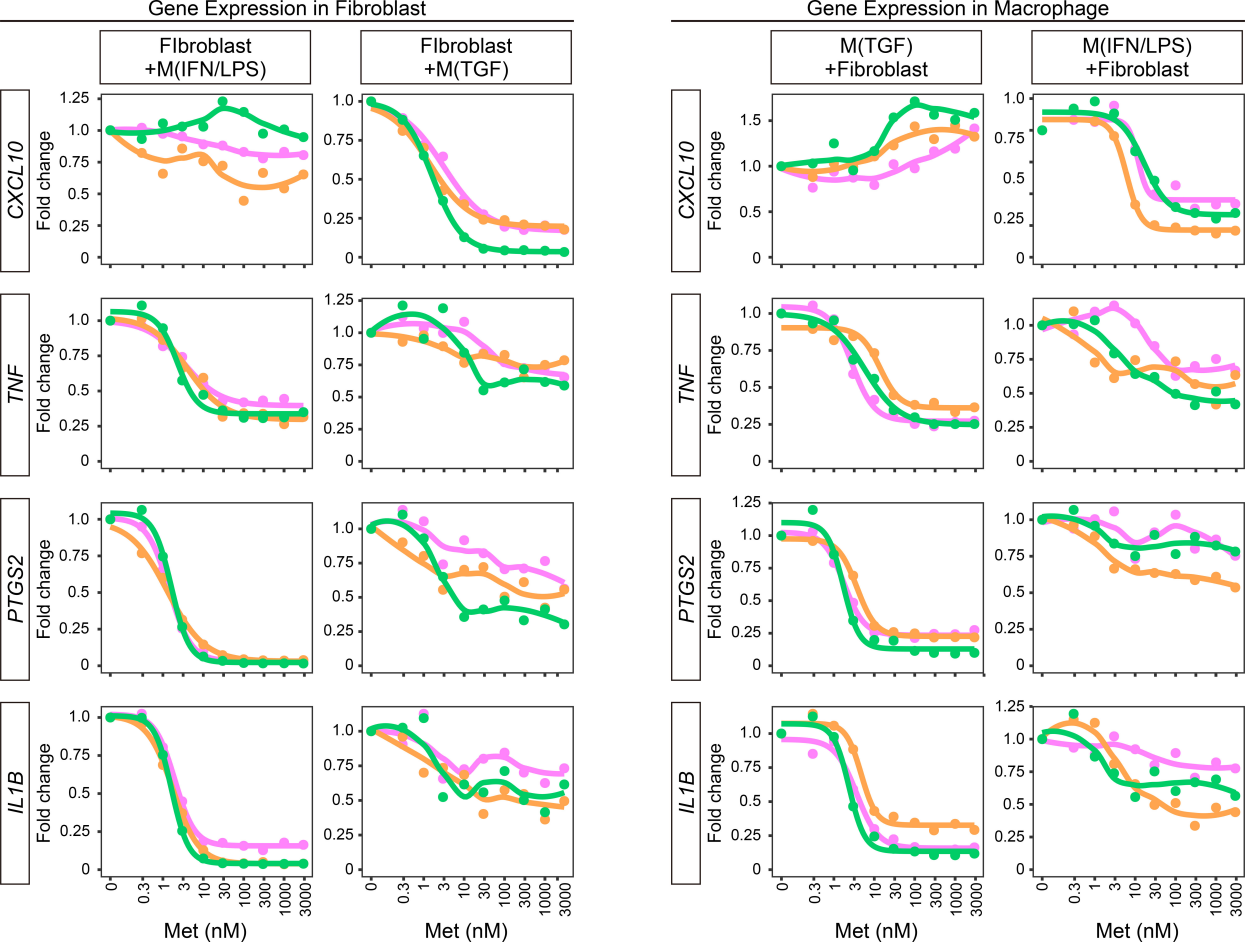
Inflammatory gene expression in fibroblast-macrophage co-culture. Human VF fibroblasts were directly co-cultured with G-M(IFN/LPS) or G-M(TGF) +/− 0.3–3,000nM methylprednisolone for 24 hours. Fibroblasts and macrophages were separated by FACS. Relative expression levels of *CXCL10*, *TNF*, *PTGS2*, and *IL1B* were determined by qPCR. Expression in cells unexposed to methylprednisolone was set to ‘1’ and fold changes in expression levels were plotted. Data were obtained from independently performed technical replicates and are illustrated in different colors. Concentration-dependent curves are also presented for data applicable to both estimation of IC_50_/EC_50_ and fitting to sigmoid curves in all triplicate experiments.

M(IFN/LPS) and M(TGF) stimulate inflammatory and fibrotic responses of VF fibroblasts.^27^ Concentration-dependent effects of methylprednisolone on gene expression were assessed using direct co-culture models of human VF fibroblasts with GFP-expressing M(IFN/LPS) and M(TGF) macrophages (G-M(IFN/LPS) and G-M(TGF)). In human VF fibroblasts co-cultured with G-M(IFN/LPS) macrophages, three inflammatory genes (*TNF, PTGS2,* and *IL1B*) were downregulated by methylprednisolone in a concentration-dependent manner. However, *CXCL10*, another inflammatory gene, was unchanged (Fig. 1). In co-culture with G-M(TGF), methylprednisolone decreased *CXCL10* expression in VF fibroblasts and tended to inhibit expression of *TNF, PTGS2,* and *IL1B*. Methylprednisolone downregulated *TNF, PTGS2,* and *IL1B* in G-M(IFN/LPS) macrophages, and *CXCL10* in G-M(TGF) macrophages in a concentration-dependent manner. In addition, *TNF, PTGS2,* and *IL1B* tended to decrease in response to methylprednisolone in G-M(TGF) macrophages. Collectively, IC_50_ and IC_90_ to decrease inflammatory genes were 1.8–3.3 and 6.8–27nM in VF fibroblasts (**Table 1**) and 2.4–11.6 and 7.7–36.8nM in macrophages (**Table 2**).

### Methylprednisolone altered fibrotic gene expression in direct co-culture of human VF fibroblasts and macrophages

**Figure 2:**
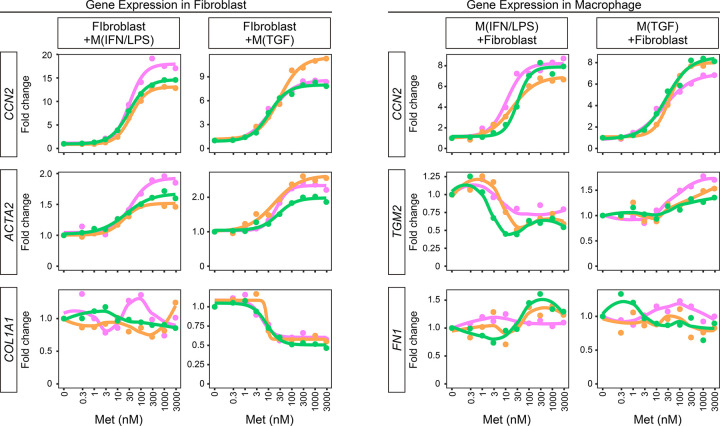
Fibrotic gene expression in fibroblast-macrophage co-culture. Human VF fibroblasts were directly co-cultured with G-M(IFN/LPS) or G-M(TGF) +/− 0.3–3,000nM methylprednisolone for 24 hours. Fibroblasts and macrophages were separated by FACS. Relative expression levels of *CCN2, ACTA2*, and *COL1A1* in fibroblasts, and *CCN2, TGM2,* and *FN1* in macrophages were determined by qPCR. Expression in cells unexposed to methylprednisolone was set to ‘1’ and fold changes in expression levels were plotted. Data were obtained from independently performed technical replicates and are illustrated in different colors. Concentration-dependent curves are also presented for data applicable to both estimation of IC_50_/EC_50_ and fitting to sigmoid curves in all triplicate experiments.

Methylprednisolone increased *CCN2* and *ACTA2* expression in VF fibroblasts directly co-cultured with G-M(IFN/LPS) and G-M(TGF) macrophages in a concentration-dependent manner (Fig. 2). However, *COL1A1* expression was unchanged or decreased by methylprednisolone. *CCN2* in G-M(IFN/LPS) and G-M(TGF) macrophages was concentration-dependently upregulated by methylprednisolone in the direct co-culture model. *TGM2* and *FN1*, M2 markers associated with fibrosis, were also analyzed.^17,27,40^ The effect on *FN1* expression was unclear. Weak tendencies of decreased and increased *TGM2* were observed in response to methylprednisolone in G-M(IFN/LPS) and G-M(TGF) macrophages, respectively. In fibroblasts, EC_50_ and EC_90_ of methylprednisolone to increase *CCN2* and *ACTA2* were 17–34 and 181–249nM; IC_50_ and IC_90_ to decrease *COL1A1* were approximately 7 and 22nM (**Table 1**). In macrophages, EC_50_ and EC_90_ to increase *CCN2* were 21–28 and 151–316nM (**Table 2**).

### Ser211-phosphorylated GR was increased by low concentration methylprednisolone

**Figure 3:**
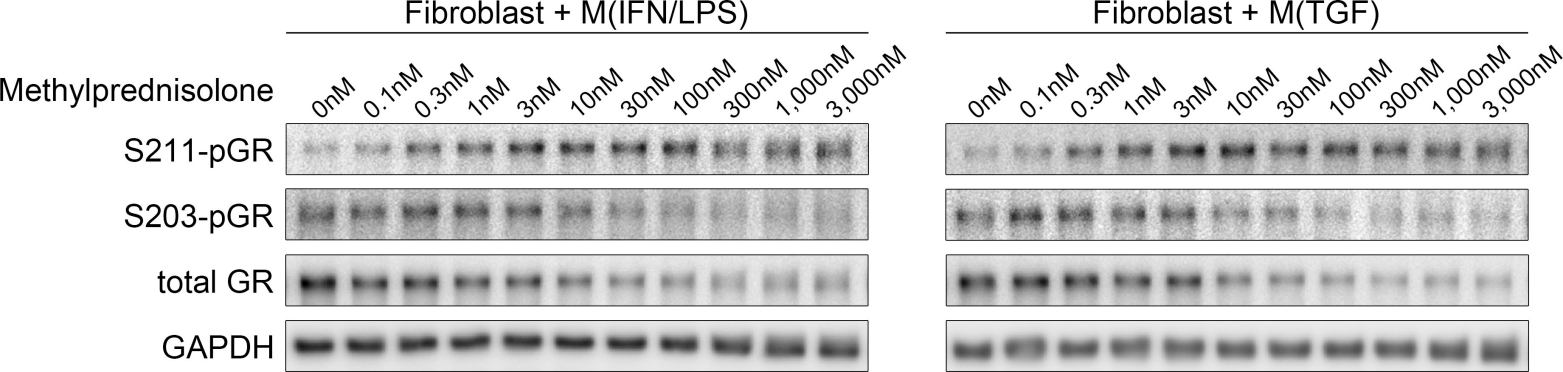
Western blots for S211-pGR, S203-pGR, and tGR. Human VF fibroblasts were directly co-cultured with M(IFN/LPS) or M(TGF) macrophages ± 0.1–3,000nM methylprednisolone for 24 hours. Proteins were extracted from the co-cultured cells without separating fibroblasts and macrophages.

Phosphorylation at Ser211 is thought to be most critical for ligand-induced activation of GR, as well as nuclear translocation.^41,42^ We performed Western blotting to assess altered Ser211-phosphorylated GR (S211-pGR), as well as total and Ser203-phosphorylated GR (tGR and S203-pGR). Directly co-cultured fibroblasts and macrophages were collected together and protein levels were assessed as mixtures of co-cultured cells. S211-pGR levels were increased by methylprednisolone regardless of macrophage phenotype and peaked around 10nM methylprednisolone (Fig. 3). This concentration was closer to the concentration required for gene downregulation than upregulation. However, S203-pGR, an inhibito of nuclear GR localization,^42^ and tGR were decreased by methylprednisolone. Similar results were observed in separately collected fibroblasts and macrophages from indirect co-culture (**Supplemental Figure S1**).

### Nuclear localization of Ser211-phosphorylated GR was increased by high concentration methylprednisolone

**Figure 4:**
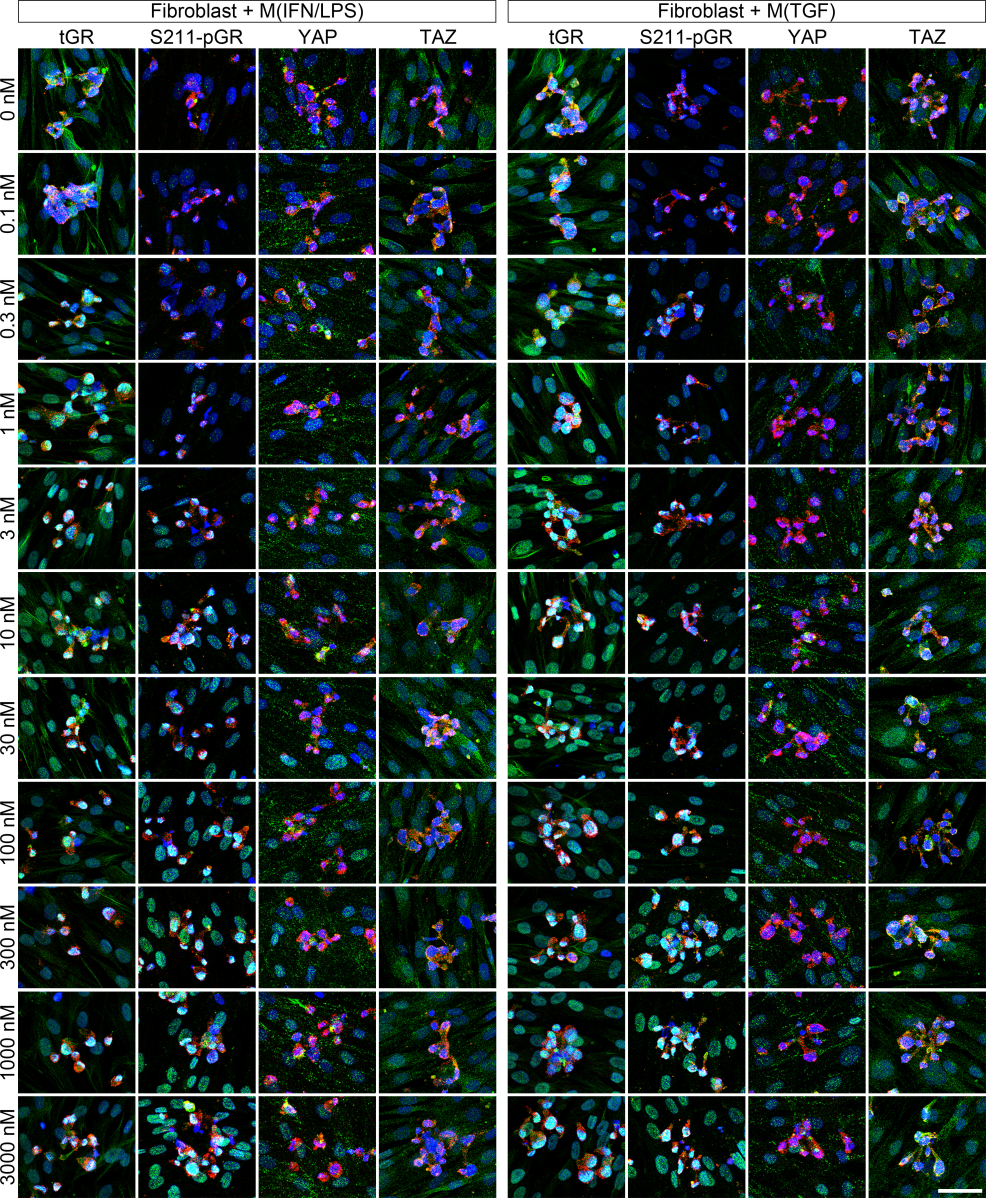
Immunocytochemistry of tGR, S211-pGR, YAP, and TAZ. Human VF fibroblasts were directly co-cultured with M(IFN/LPS) or M(TGF) macrophages ± 0.1–3,000nM methylprednisolone for 24 hours. Green: tGR, S211-pGR, YAP, and TAZ. Red: Macrophages (CD11B and F4/80). Blue: Nuclei (DAPI). Bar: 50μm.

We subsequently performed immunocytochemistry to assess nuclear localization of GR. Regardless of macrophage phenotype, tGR-positive staining in DAPI-positive nuclear regions was concentration-dependently increased by methylprednisolone in both fibroblasts and macrophages in co-culture. tGR staining in nuclear regions peaked at 3–30nM methylprednisolone and decreased at higher concentrations (Fig. 4). S211-pGR in nuclear regions was also increased by methylprednisolone and plateaued at 100–300nM methylprednisolone. These findings, collectively with qPCR data, suggested the concentration of methylprednisolone required to upregulate fibrotic genes was more related to S211-pGR nuclear localization than tGR.

#### Methylprednisolone induced Nuclear localization of YAP/TAZ in VF fibroblasts.

Distribution of YAP/TAZ was assessed by immunocytochemistry. Regardless of the phenotype of co-cultured macrophages, positive staining for YAP and TAZ increased in the nucleus of VF fibroblasts as methylprednisolone concentrations increased (Fig. 4). In contrast, methylprednisolone did not alter the distribution of YAP/TAZ in M(IFN/LPS) or M(TGF) macrophages.

#### Fibrotic gene expression induced by methylprednisolone was suppressed by inhibition of YAP/TAZ-TEAD signaling.

**Figure 5:**
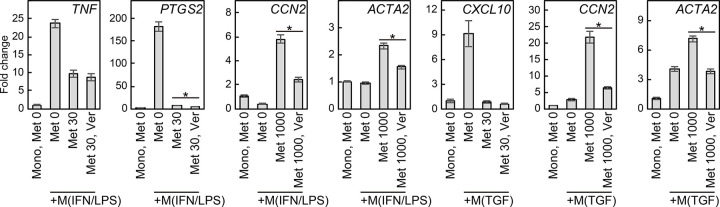
Effects of pharmacological YAP/TAZ-TEAD inhibition on methylprednisolone-induced gene regulation. Human VF fibroblasts were directly co-cultured with G-M(IFN/LPS) or G-M(TGF) macrophages +/− 30 or 1,000nM methylprednisolone and +/− 3μM verteporfin for 24 hours. Fibroblasts were isolated by FACS. Relative expression levels of *TNF, PTGS2, CCN2,* and *ACTA2* in fibroblasts co-cultured with G-M(IFN/LPS) macrophages, and *CXCL10, CCN2,* and *ACTA2* in fibroblasts co-cultured with G-M(TGF) macrophages were determined by qPCR. Expression in mono-cultured fibroblasts was set to ‘1.’ Data are presented as mean ± SD. Co-cultured fibroblasts exposed to only methylprednisolone, and both methylprednisolone and verteporfin were subjected to statistical consideration with Student’s *t*-test. Statistically significant differences are denoted as asterisks.

To assess involvement of YAP/TAZ in the negative and positive gene regulation of methylprednisolone, we pharmacologically inhibited YAP/TAZ-TEAD signaling in co-cultured fibroblasts in the presence of 30 or 1,000nM methylprednisolone. In VF fibroblasts co-cultured with M(IFN/LPS) macrophages, the decrease of *TNF* and *PTGS2* by 30nM methylprednisolone was not reversed by verteporfin (Fig. 5). However, increased *CCN2*, a target gene of YAP/TAZ-TEAD signaling, induced by 1,000nM methylprednisolone was inhibited by verteporfin. Verteporfin also inhibited *ACTA2*. Similarly, *CXCL10* downregulation by 30nM methylprednisolone was not prevented by verteporfin in VF fibroblasts co-cultured with M(TGF) macrophages, whereas increased *CCN2* and *ACTA2* induced by 1,000nM methylprednisolone was ameliorated by verteporfin. These findings suggested *CCN2* upregulation mediated by high-concentration GCs was driven with support of YAP/TAZ-TEAD signaling, but GC-induced suppression of those inflammatory genes was independent of YAP/TAZ-TEAD signaling.

## DISCUSSION

GC therapy persists as a reasonable therapeutic option due to potent anti-inflammatory effects as well as the affordability and safety data accumulated over decades of clinical use. However, GCs have diverse functions and unfavorable side effects may negatively affect clinical outcomes. Optimizing GC therapy by reducing unfavorable effects could benefit millions of patients, while developing a novel therapeutic would also advance clinical care. The current study provided incremental data regarding concentration-dependent negative and positive gene regulation by methylprednisolone, as well as insight regarding mechanism(s) underlying this effect.

The IC_90_ of methylprednisolone to reduce inflammatory gene transcription was lower than the EC_90_ to promote fibrotic transcription in the current direct co-culture model; these data are similar to mono-cultured and indirectly co-cultured macrophages and VF fibroblasts.^30,31^ This finding further supports our hypothesis that reduced GC concentrations to a level sufficient to inhibit inflammatory response is preferable to minimize fibrotic side effects.^15^ As noted in the previous indirect co-culture study,^31^ the IC_90_ range of methylprednisolone in the co-culture model was comparable to peak plasma levels of free methylprednisolone (21.1nM) after oral administration,^43,44^ but the EC_90_ values were lower than the concentration of methylprednisolone for injection (53–214mM).^45^ *In vivo* investigation is warranted to optimize GC dosing for efficiently inhibiting inflammation without activating fibrotic response.

The GR response to different concentrations of methylprednisolone was complex. Our data suggest concentration-dependent differential effects on GR phosphorylation/nuclear localization contribute to the complex biochemistry of GC-GR signaling. Although our data are insufficient to detail molecular events associated with different concentrations of GCs, some similarities were observed between methylprednisolone concentrations required to alter GR status and gene expression. Ideally, these data may be foundational to unravel the complexity related to GC concentration. Concentrations required to fully increase nuclear localization of S211-pGR were 100–300nM, comparable to the EC_90_ for upregulation of fibrotic genes. In contrast, S211-pGR and nuclear localization of tGR reached a peak at 3–30nM methylprednisolone, close to the IC_90_ for gene downregulation. Therefore, it seems the nuclear level of GR without Ser211-phosphorylation and/or cytoplasmic S211-pGR is related to negative gene regulation via low GC concentration, and the nuclear localization of S211-pGR is related to positive gene regulation via high GC concentration. Nuclear localization of GR and its recruitment to positive gene regulatory elements are restricted by various types of post-translational modifications. These phenomena may explain why increased S211-pGR by low concentration methylprednisolone did not lead to increased nuclear accumulation or *CCN2* upregulation. For example, phosphorylation at Ser203 and Ser226, and acetylation at Lys494 and Lys495 prevent nuclear localization and/or recruitment to positive gene regulatory elements.^10,42,46^ Regarding Ser203 phosphorylation, the function of Ser211 phosphorylation to drive nuclear localization of GR is likely overcome by Ser203 phosphorylation to prevent nuclear localization of GR.^42^ On the other hand, recruitment of GR to negative gene regulatory elements, presumably associated with low-concentration methylprednisolone, reportedly requires SUMOylation at Lys293 of GR.^47^ In addition, concentration of ligand-activated GR in the nucleus is thought to be crucial for GR dimerization and GR-mediated transcription via positive gene regulatory elements.^10,48,49^ In spite of our encouraging data, mechanisms underlying concentration-dependent negative and positive gene regulation are still unclear.

YAP/TAZ-TEAD signaling supports multiple fibrotic signaling pathways, such as SMAD, Wnt, and Rho.^50^ Inhibition of YAP/TAZ-TEAD signaling, as well as neutralization of connective tissue growth factor (encoded by *CCN2*), reduced the fibrotic response in multiple animal models and VF fibroblasts.^35,36,51,52^ In the current study, *CCN2* upregulation induced by high-concentration methylprednisolone was reversed by verteporfin, but downregulation of *CXCL10, TNF,* and *PTGS2* was not. This finding suggests the fibrotic response associated with YAP/TAZ-TEAD signaling is specifically induced by high-concentration GCs. Blocking YAP/TAZ in combination with GC therapy may be another possible strategy to reduce fibrotic response induced by GCs. However, with regard to inflammation, YAP/TAZ reportedly has positive and negative roles dependent on cell types and organs.^50,53^ Notably, as shown in our immunocytochemistry results, YAP/TAZ and TEAD activities in hematopoietic cells are quite different from other cells.^50^ The impact of YAP/TAZ and TEADs in inflammatory responses of VF-resident and hematopoietic cells requires investigation to potentially target YAP/TAZ for VF diseases.

In conclusion, concentration-dependent differential effects of methylprednisolone were broadly observed across *in vitro* co-culture models of human VF fibroblasts and macrophages. S211-pGR nuclear localization and the activation of YAP/TAZ-TEAD signaling were likely associated with fibrotic gene expression mediated by high-concentration methylprednisolone in VF fibroblasts.

## MATERIALS AND METHODS

The Supporting Information provides more detailed methodological specifics.

### Cells.

HVOX human VF fibroblasts, created by our group,^54^ and THP-1 human monocytic cells (ATCC, Manassas, VA) were expanded as described previously.^36^ Green fluorescent protein (GFP)-expressing THP-1 cells were prepared by transfection of pAcGFP1-Actin (Takara Bio, Shiga, Japan). M(IFN/LPS) and M(TGF) macrophages were prepared by stimulating THP-1-derived macrophages with IFN-γ/LPS, and TGF-β, and directly co-cultured with fibroblasts as described previously.^27^ Cells were exposed to different concentrations of methylprednisolone and/or 3μM verteporfin for 24 hours.

### Quantitative Real-Time Polymerase Chain Reaction (qPCR).

HVOX fibroblasts and GFP-positive macrophages were separated by fluorescence-activated cell sorting (FACS). RNA extraction, reverse transcription, and real-time polymerase chain reaction were performed using commercially available kits. Expression levels relative to *GAPDH* were quantified by the ΔΔCt method.

### Western blotting and Immunocytochemistry.

Following co-culture of fibroblasts and GFP-negative macrophages, Western blotting and immunocytochemistry were performed as described previously.^27,37^ Antibodies are shown in **Table S1.**

### Data analysis.

Data were collected from independently performed technical triplicate experiments, at least. The R *drc* package was employed on R studio to determine EC_50_, IC_50_, EC_90_, and IC_90_, and to fit data into sigmoid curves, when applicable.^55^ Simple dot plots are presented for other data. Means of EC_x_/IC_x_, as well as standard deviations, were calculated from the EC_x_/IC_x_ estimations of triplicate experiments.

## Data Availability

All data supporting the findings of this study are available from the corresponding author upon reasonable request.

## References

[1] Titze IR, Hunter EJ. Normal vibration frequencies of the vocal ligament. J Acoust Soc Am 2004; 115:2264–2269. 15139637 10.1121/1.1698832 PMC1552154

[2] Cohen SM, Kim JW, Roy N, Asche C, Courey M. Direct health care costs of laryngeal diseases and disorders. Laryngoscope 2012; 122:1582–1588. 22544473 10.1002/lary.23189

[3] Mortensen M, Woo P. Office steroid injections of the larynx. Laryngoscope 2006; 116:1735–1739. 17003727 10.1097/01.mlg.0000231455.19183.8c

[4] Woo J-H, Kim D-Y, Kim J-W, Oh E-A, Lee S-W. Efficacy of percutaneous vocal fold injections for benign laryngeal lesions: Prospective multicenter study. Acta Oto-Langologica 2011; 22:1326–1330. 10.3109/00016489.2011.620620 22074107

[5] Lee S-H, Yeo J-O, Choi J-I Local steroid injection via the cricothyroid membrane in patients with a vocal nodule. Arch Otolaryngol Head Neck Surg 2011; 137:1011–1016. 22006779 10.1001/archoto.2011.168

[6] Cain DW, Cidlowski JA. Immune regulation by glucocorticoids. Nat Rev Immunol 2017; 17:233–247. 28192415 10.1038/nri.2017.1 PMC9761406

[7] Govil N, Paul BC, Amin MR, Branski RC. The utility of glucocorticoids for vocal fold pathology: A survey of otolaryngologists. Journal of Voice 2014; 28:82–87. 24050821 10.1016/j.jvoice.2013.04.015

[8] Takahashi S, Kanazawa T, Hasegawa T Comparison of therapeutic effects of steroid injection by benign vocal fold lesion type. Acta Otolaryngol 2021; 141:1005–1013. 34751085 10.1080/00016489.2021.1995895

[9] Young WG, Hoffman MR, Koszewski IJ, Whited CW, Ruel BN, Dailey SH. Voice Outcomes following a Single Office-Based Steroid Injection for Vocal Fold Scar. Otolaryngol Head Neck Surg 2016; 155:820–828. 27507145 10.1177/0194599816654899

[10] Weikum ER, Knuesel MT, Ortlund EA, Yamamoto KR. Glucocorticoid receptor control of transcription: precision and plasticity via allostery. Nat Rev Mol Cell Biol 2017; 18:159–174. 28053348 10.1038/nrm.2016.152 PMC6257982

[11] Petta I, Dejager L, Ballegeer M The Interactome of the Glucocorticoid Receptor and Its Influence on the Actions of Glucocorticoids in Combatting Inflammatory and Infectious Diseases. Microbiol Mol Biol Rev 2016; 80:495–522. 27169854 10.1128/MMBR.00064-15 PMC4867367

[12] Ismaili N, Garabedian MJ. Modulation of glucocorticoid receptor function via phosphorylation. Ann N Y Acad Sci 2004; 1024:86–101. 15265775 10.1196/annals.1321.007

[13] Langenbach SY, Wheaton BJ, Fernandes DJ Resistance of fibrogenic responses to glucocorticoid and 2-methoxyestradiol in bleomycin-induced lung fibrosis in mice. Can J Physiol Pharmacol 2007; 85:727–738. 17823636 10.1139/Y07-065

[14] Okada H, Kikuta T, Inoue Tet al. Dexamethasone induces connective tissue growth factor expression in renal tubular epithelial cells in a mouse strain-specific manner. Am J Pathol 2006; 168:737–747. 16507889 10.2353/ajpath.2006.050656 PMC1606512

[15] Nakamura R, Mukudai S, Bing R, Garabedian MJ, Branski RC. Complex fibroblast response to glucocorticoids may underlie variability of clinical efficacy in the vocal folds. Sci Rep 2020; 10:20458. 33235235 10.1038/s41598-020-77445-9 PMC7686477

[16] Kendall RT, Feghali-Bostwick CA. Fibroblasts in fibrosis: novel roles and mediators. Front Pharmacol 2014; 5:123. 24904424 10.3389/fphar.2014.00123 PMC4034148

[17] Biswas SK, Mantovani A. Macrophages: Biology and Role in the Pathology of Diseases. Berlin, Germany: Springer, 2014.

[18] Wynn TA, Ramalingam TR. Mechanisms of fibrosis: therapeutic translation for fibrotic disease. Nat Med 2012; 18:1028–1040. 22772564 10.1038/nm.2807 PMC3405917

[19] Kaba S, Nakamura R, Yamashita M Alterations in macrophage polarization in injured murine vocal folds. Laryngoscope 2019; 129:E135–E142. 30597576 10.1002/lary.27523

[20] Kaba S, Kawai Y, Tanigami Y Peroxisome Proliferator-Activated Receptor-gamma Agonist Attenuates Vocal Fold Fibrosis in Rats via Regulation of Macrophage Activation. Am J Pathol 2022; 192:771–782. 35189097 10.1016/j.ajpath.2022.02.002

[21] Biswas SK, Mantovani A. Macrophage plasticity and interaction with lymphocyte subsets: cancer as a paradigm. Nat Immunol 2010; 11:889–896. 20856220 10.1038/ni.1937

[22] Braga TT, Agudelo JS, Camara NO. Macrophages During the Fibrotic Process: M2 as Friend and Foe. Front Immunol 2015; 6:602. 26635814 10.3389/fimmu.2015.00602 PMC4658431

[23] Spiller KL, Wrona EA, Romero-Torres S Differential gene expression in human, murine, and cell line-derived macrophages upon polarization. Exp Cell Res 2016; 347:1–13. 26500109 10.1016/j.yexcr.2015.10.017

[24] Wynn TA, Vannella KM. Macrophages in Tissue Repair, Regeneration, and Fibrosis. Immunity 2016; 44:450–462. 26982353 10.1016/j.immuni.2016.02.015 PMC4794754

[25] Zhang F, Wang H, Wang X TGF-β induces M2-like macrophage polarization via SNAIL-mediated suppression of a pro-inflammatory phenotype. Oncotarget 2016; 7:52294–52306. 27418133 10.18632/oncotarget.10561 PMC5239552

[26] Gordon S, Martinez-Pomares L. Physiological roles of macrophages. Pflugers Arch 2017; 469:365–374. 28185068 10.1007/s00424-017-1945-7 PMC5362657

[27] Nakamura R, Bing R, Gartling GJ, Branski RC. Macrophages alter inflammatory and fibrotic gene expression in human vocal fold fibroblasts. Exp Cell Res 2022; 419:113301. 35931141 10.1016/j.yexcr.2022.113301

[28] Sheng J, Yang Y, Cui Yet al. M2 macrophage-mediated interleukin-4 signalling induces myofibroblast phenotype during the progression of benign prostatic hyperplasia. Cell Death Dis 2018; 9:755. 29988032 10.1038/s41419-018-0744-1 PMC6037751

[29] Ding Q, Sun J, Xie W, Zhang M, Zhang C, Xu X. Stemona alkaloids suppress the positive feedback loop between M2 polarization and fibroblast differentiation by inhibiting JAK2/STAT3 pathway in fibroblasts and CXCR4/PI3K/AKT1 pathway in macrophages. Int Immunopharmacol 2019; 72:385–394. 31030094 10.1016/j.intimp.2019.04.030

[30] Nakamura R, Bing R, Gartling GJ, Garabedian MJ, Branski RC. Glucocorticoid Dose Dependency on Gene Expression in Vocal Fold Fibroblasts and Macrophages. Laryngoscope 2023; 133:1169–1175. 36779842 10.1002/lary.30330 PMC9925845

[31] Nakamura R, Bing R, Gartling GJ, Garabedian MJ, Branski RC. Concentration Effects of Methylprednisolone in Human Vocal Fold Fibroblast-Macrophage Co-Culture. Laryngoscope 2023; 133:3116–3122. 37246727 10.1002/lary.30763 PMC10592568

[32] Lodyga M, Cambridge E, Karvonen HM Cadherin-11-mediated adhesion of macrophages to myofibroblasts establishes a profibrotic niche of active TGF-β. Sci Signal 2019; 12. 10.1126/scisignal.aao3469 30647145

[33] Nakamura R, Bing R, Gartling GJ, Garabedian MJ, Branski RC. Dose-Dependent Glucocorticoid Regulation of Transcription Factors in Vocal Fold Fibroblasts and Macrophages. Laryngoscope 2023; 133:2704–2711. 36752581 10.1002/lary.30594 PMC10406972

[34] Grannas K, Arngarden L, Lonn P Crosstalk between Hippo and TGFbeta: Subcellular Localization of YAP/TAZ/Smad Complexes. J Mol Biol 2015; 427:3407–3415. 25937570 10.1016/j.jmb.2015.04.015

[35] Seo E, Kim WY, Hur J The Hippo-Salvador signaling pathway regulates renal tubulointerstitial fibrosis. Sci Rep 2016; 6:31931. 27550469 10.1038/srep31931 PMC4994041

[36] Nakamura R, Hiwatashi N, Bing R, Doyle CP, Branski RC. Concurrent YAP/TAZ and SMAD signaling mediate vocal fold fibrosis. Sci Rep 2021; 11:13484. 34188130 10.1038/s41598-021-92871-z PMC8241934

[37] Nakamura R, Bing R, Doyle CP, Garabedian MJ, Branski RC. Glucocorticoids activate Yes-associated protein in human vocal fold fibroblasts. Exp Cell Res 2021; 405:112681. 34087241 10.1016/j.yexcr.2021.112681 PMC8328950

[38] Fu V, Plouffe SW, Guan KL. The Hippo pathway in organ development, homeostasis, and regeneration. Curr Opin Cell Biol 2017; 49:99–107. 29316535 10.1016/j.ceb.2017.12.012 PMC6348871

[39] Lai D, Ho KC, Hao Y, Yang X. Taxol resistance in breast cancer cells is mediated by the hippo pathway component TAZ and its downstream transcriptional targets Cyr61 and CTGF. Cancer Res 2011; 71:2728–2738. 21349946 10.1158/0008-5472.CAN-10-2711

[40] Hoeft K, Schaefer GJL, Kim H Platelet-instructed SPP1(+) macrophages drive myofibroblast activation in fibrosis in a CXCL4-dependent manner. Cell Rep 2023; 42:112131. 36807143 10.1016/j.celrep.2023.112131 PMC9992450

[41] Khan SH, McLaughlin WA, Kumar R. Site-specific phosphorylation regulates the structure and function of an intrinsically disordered domain of the glucocorticoid receptor. Sci Rep 2017; 7:15440. 29133811 10.1038/s41598-017-15549-5 PMC5684351

[42] Wang Z, Frederick J, Garabedian MJ. Deciphering the phosphorylation “code” of the glucocorticoid receptor in vivo. J Biol Chem 2002; 277:26573–26580. 12000743 10.1074/jbc.M110530200

[43] Rohatagi S, Barth J, Möllmann H Pharmacokinetics of methylprednisolone and prednisolone after single and multiple oral administration. J Clin Pharmacol 1997; 37:916–925. 10.1002/j.1552-4604.1997.tb04266.x 9505983

[44] Feingold KR, Anawalt B, Boyce Aet al. Endotext, 2000.

[45] DrugBank Online. Available at: https://go.drugbank.com. Accessed June 15th 2022.

[46] Kino T, Chrousos GP. Acetylation-mediated epigenetic regulation of glucocorticoid receptor activity: circadian rhythm-associated alterations of glucocorticoid actions in target tissues. Mol Cell Endocrinol 2011; 336:23–30. 21146585 10.1016/j.mce.2010.12.001 PMC3057275

[47] Hua G, Ganti KP, Chambon P. Glucocorticoid-induced tethered transrepression requires SUMOylation of GR and formation of a SUMO-SMRT/NCoR1-HDAC3 repressing complex. Proc Natl Acad Sci U S A 2016; 113:E635–643. 26712006 10.1073/pnas.1522826113 PMC4747779

[48] Robertson S, Rohwer JM, Hapgood JP, Louw A. Impact of glucocorticoid receptor density on ligand-independent dimerization, cooperative ligand-binding and basal priming of transactivation: a cell culture model. PLoS One 2013; 8:e64831. 23717665 10.1371/journal.pone.0064831 PMC3661511

[49] Louw A. GR Dimerization and the Impact of GR Dimerization on GR Protein Stability and Half-Life. Front Immunol 2019; 10:1693. 31379877 10.3389/fimmu.2019.01693 PMC6653659

[50] Ma S, Meng Z, Chen R, Guan KL. The Hippo Pathway: Biology and Pathophysiology. Annu Rev Biochem 2019; 88:577–604. 30566373 10.1146/annurev-biochem-013118-111829

[51] Ikawa Y, Ng PS, Endo K Neutralizing monoclonal antibody to human connective tissue growth factor ameliorates transforming growth factor-beta-induced mouse fibrosis. J Cell Physiol 2008; 216:680–687. 18481257 10.1002/jcp.21449

[52] Mannaerts I, Leite SB, Verhulst S The Hippo pathway effector YAP controls mouse hepatic stellate cell activation. J Hepatol 2015; 63:679–688. 25908270 10.1016/j.jhep.2015.04.011

[53] Wang S, Zhou L, Ling L The Crosstalk Between Hippo-YAP Pathway and Innate Immunity. Front Immunol 2020; 11:323. 32174922 10.3389/fimmu.2020.00323 PMC7056731

[54] Branski RC, Barbieri SS, Weksler BB Effects of transforming growth factor-beta1 on human vocal fold fibroblasts. Ann Otol Rhinol Laryngol 2009; 118:218–226. 19374154 10.1177/000348940911800310

[55] Ritz C, Baty F, Streibig JC, Gerhard D. Dose-Response Analysis Using R. PLoS One 2015; 10:e0146021. 26717316 10.1371/journal.pone.0146021 PMC4696819

